# Patterns of Biflavonoid Accumulation in Ginkgo (*Ginkgo biloba* L.) Leaves from 90 Trees and Their Variation with Age, Gender, and Location

**DOI:** 10.3390/plants15111724

**Published:** 2026-06-02

**Authors:** Dunja Šamec, Barbara Medvedec, Iva Jurčević Šangut, Ana Jurinjak Tušek

**Affiliations:** 1 Department of Food Technology, University North, Trg Dr. Žarka Dolinara 1, 48 000 Koprivnica, Croatia; bmedvedec@unin.hr (B.M.); ijurcevic@unin.hr (I.J.Š.); 2 Faculty of Food Technology and Biotechnology, University of Zagreb, Pierottijeva 6, 10 000 Zagreb, Croatia; ana.tusek.jurinjak@pbf.unizg.hr

**Keywords:** biflavonoids, ginkgo, sciadopitysin

## Abstract

Biflavonoids are dimeric flavonoids recognized for their diverse biological activities and significant pharmacological potential, with ginkgo (*Ginkgo biloba* L.) serving as a primary natural source. This study presents a comprehensive spatiotemporal characterization of the biflavonoid profile across a diverse population of 90 trees. High-resolution chromatographic analysis quantified five major biflavonoids, revealing a consistent hierarchical abundance: sciadopitysin > isoginkgetin > ginkgetin > bilobetin > amentoflavone. Notably, sciadopitysin emerged as the predominant constituent (1532.89 ± 544.13 µg/g dw). To decode the complex drivers of metabolite accumulation, we integrated Principal Component Analysis (PCA) with Piecewise Linear Regression (PLR). PCA confirmed a robust chemical structure, explaining 71.5% of the total variance, where Factor 1 represents a general biflavonoid gradient and Factor 2 captures localized environmental influences. The PLR models (*R*^2^ = 0.75–0.83) identified tree age as a primary negative regulator, showing a significant decline in total biflavonoids as trees mature beyond the 30-year reproductive threshold. While sexual dimorphism and location exhibited compound-specific nonlinear effects, younger trees (10–30 years) demonstrated the highest biosynthetic plasticity and potency. These findings establish a predictive framework for optimizing the pharmaceutical harvest of ginkgo leaves, highlighting that age-related physiological shifts, rather than gender or broad geography, are the critical determinants of biflavonoids yield.

## 1. Introduction

Biflavonoids are dimeric flavonoids composed of two monomeric flavonoid subunits linked by either carbon–carbon or carbon–oxygen bonds [1,2]. Although monomeric flavonoids have attracted considerable scientific attention as bioactive compounds, biflavonoids have been comparatively underexplored. Nevertheless, in recent years, numerous studies have highlighted their diverse biological activities, including antioxidant, anti-inflammatory, antiviral, antibacterial, neuroprotective, anticancer, and cardioprotective effects, as well as enzyme inhibitory activities [3,4,5,6].

The first biflavonoid, ginkgetin, was isolated from yellow leaves of ginkgo (*Ginkgo biloba* L.) approximately one hundred years ago. Since then, a wide variety of biflavonoid structures have been reported in the literature [2]. However, some older reports require careful re-evaluation due to historically unstandardized nomenclature and structural characterization methods, which have led to possible misidentification or the reporting of identical compounds under different names [7]. According to current literature, approximately 600 distinct biflavonoid structures have been described, although this number may vary depending on how these inconsistencies are resolved [1]. In ginkgo, thirteen different biflavonoids have been reported, including both aglycones and glycosylated derivatives [8]. Among these, five compounds are consistently described as the most abundant and most frequently detected: amentoflavone, bilobetin, ginkgetin, isoginkgetin, and sciadopitysin [8,9]. However, discrepancies remain in the literature; for example, some studies do not report the presence of amentoflavone [10], while others describe ginkgetin and isoginkgetin as a single compound [11]. Recent studies have identified genes potentially involved in biflavonoid biosynthesis in ginkgo [12], yet the regulatory mechanisms and environmental or physiological factors influencing their accumulation remain largely unknown. It has been suggested that biflavonoid content may vary depending on the developmental stage of the leaves. Wang et al. [10] investigated the levels of ginkgetin, isoginkgetin, bilobetin, and sciadopitysin in ginkgo leaves across six developmental stages, reporting biflavonoid contents ranging from 0 to 800 ng/g with significant variation depending on leaf development. Sciadopitysin was detected in all samples, with the highest concentrations observed at more advanced developmental stages. Similarly, bilobetin and ginkgetin reached their highest levels in the final developmental stage, whereas isoginkgetin was most abundant in the youngest leaves. In our previous study [13], we also examined biflavonoid content in ginkgo leaves collected monthly from May to November. We observed higher biflavonoid levels in yellow leaves, with a pronounced increase in accumulation beginning in August. Biflavonoid content remained high until leaf senescence. Interestingly, biflavonoids were also detected in fallen ginkgo leaves, in some cases at even higher concentrations than in yellow leaves collected directly from the tree [14]. Plant age also appears to influence biflavonoid accumulation. Pandey et al. [15] reported that 175-year-old ginkgo trees accumulated significantly higher levels of sciadopitysin in leaves and stems compared to 8-year-old trees, but they just examined few trees per group. In another study, Kaur et al. [16] analyzed the content of bilobetin, ginkgetin, and sciadopitysin in ginkgo leaves collected from six locations in India using reverse-phase high-performance thin-layer chromatography (RP-HPTLC). Ginkgetin and sciadopitysin were detected in all samples, with ginkgetin being the most abundant compound in five out of six locations. Bilobetin was present only in samples from three locations. The authors suggested that these differences may be related to growth altitude and cultivation conditions, noting that field-grown plants tended to accumulate higher levels of bioactive constituents than naturally growing older trees. Despite these observations, the mechanisms governing biflavonoid accumulation and the influence of external factors remain poorly understood. One possible reason is that physiological studies on ginkgo flavonoids have historically focused almost exclusively on monomeric flavonoids, largely neglecting biflavonoids [17,18,19,20,21]. Similarly, studies investigating the effects of environmental factors, such as temperature, light intensity, UV-B radiation, and salt stress [22,23,24,25,26] on ginkgo flavonoid profiles have reported only monomeric flavonoid data.

In light of the increasing interest in the biological activity of biflavonoids and their potential pharmaceutical applications, understanding the factors that influence their accumulation in plant tissues has become increasingly important. However, the environmental and biological determinants of biflavonoid content in ginkgo remain poorly understood. To address these limitations, this study utilizes chromatographic approach combined with multivariate modeling to analyze a nationally representative population of 90 trees across Croatia. By evaluating the simultaneous influence of location, tree age, and gender, we aim to provide an evaluation framework for biflavonoid accumulation, offering new insights for both plant physiology and the optimization of pharmacological raw materials.

## 2. Results

### 2.1. Presence of Biflavonoids in Analysed Trees

In our study, a total of 90 leaf samples were analyzed, and the maximum, minimum, mean values, and standard deviations are presented in Table 1.

Bilobetin, ginkgetin, isoginkgetin, and sciadopitysin were detected in all 90 analyzed samples, whereas amentoflavone was quantified in only 67.8% of samples. This lower detection rate for amentoflavone (61/90) is likely attributable to its function as a primary biosynthetic precursor, leading to trace-level steady-state concentrations that frequently fell below the limit of quantitation. A robust hierarchical concentration trend was identified across the population: amentoflavone < bilobetin < ginkgetin < isoginkgetin < sciadopitysin. Sciadopitysin was detected as the most abundant biflavonoids in most of our samples. We observed an increased isoginkgetin content compared to sciadopitysin in only two of the 90 samples; in both cases, these were young ginkgo trees, one younger than 10 years and one between 10 and 30 years old. In all other samples, the biflavonoid pattern followed the sequence described above. Analysis of the coefficient of variation (CV) provided critical insights into the stability of the biflavonoid profile. Sciadopitysin exhibited the lowest variability (CV = 35.49%), suggesting that its accumulation is tightly regulated or reaches a saturated physiological plateau across different environments. In contrast, amentoflavone (CV = 94.88%) and bilobetin (CV = 81.91%) demonstrated extreme heterogeneity. Such high variance indicates that these specific compounds are highly sensitive to microenvironmental fluctuations or localized physiological stressors. The total biflavonoid content showed a wide dynamic range, spanning from 1314.74 to 11,517.60 µg/g dw. Despite this nearly 9-fold difference between the minimum and maximum specimens, the CV of 44.66% for total content reflects a relatively stable aggregate metabolic output across the diverse Croatian population.

### 2.2. Differences in Biflavonoid Content According to Tree Age, Location, and Gender

**Figure 1:**
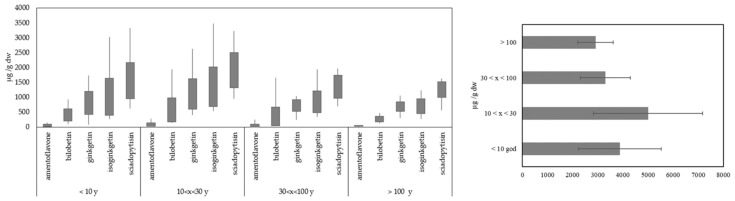
(**Left**): Biflavonoid content in ginkgo leaves across four age categories: <10 years, 10–30 years, 30–100 years, and >100 years. Data are presented as minimum, maximum, and mean values for each age group. (**Right**): Average value of total biflavonoids across four age categories: <10 years, 10–30 years, 30–100 years, and >100 years.

To examine differences across age groups, the samples were classified into four categories: younger than 10 years, 10–30 years, 30–100 years, and older than 100 years. The average, maximum, and minimum values for each group are shown in the plots in Figure 1.

As shown in Figure 1, younger trees exhibit greater variability and slightly higher concentrations of biflavonoids. This trend is also reflected in average total biflavonoid content; trees aged 10–30 years display higher levels, likely corresponding to a period of intensive growth and preparation for reproduction, which in ginkgo typically occurs after 30 years of age.

**Figure 2:**
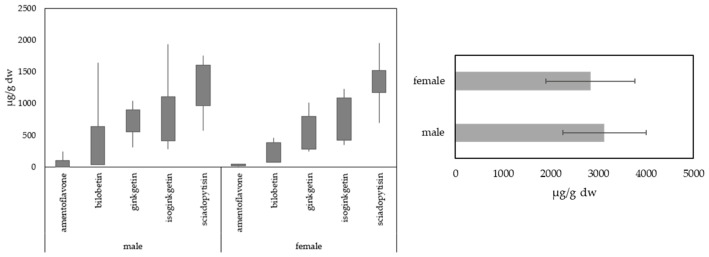
(**Left**): Content of individual biflavonoids in ginkgo leaves collected from female and male trees. Data are presented as minimum, maximum, and mean values for each age group. (**Right**): Average value of total biflavonoids in ginkgo leaves collected from female and male trees.

In our samples, tree gender was determined when possible. As ginkgo trees typically express their sex after approximately 30 years of age, only trees older than 30 years for which gender could be clearly identified were included in the gender-based analysis. This subset consisted of 23 male and 11 female trees. The average, maximum, and minimum values for each gender group are presented in Figure 2. As shown, no clear differences between male and female trees could be observed based solely on the average value.

**Figure 3:**
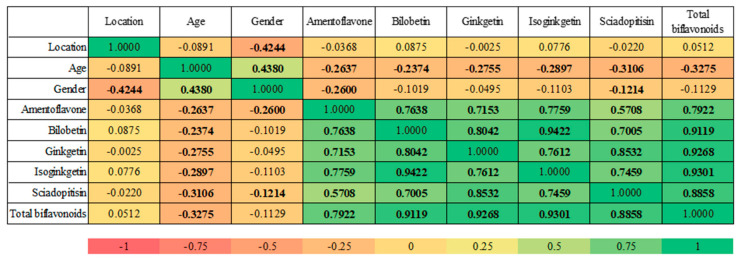
Spearman correlation matrix for analysis of the selected variables relationships. Significant correlations for *p* < 0.05 are marked bold.

**Figure 4:**
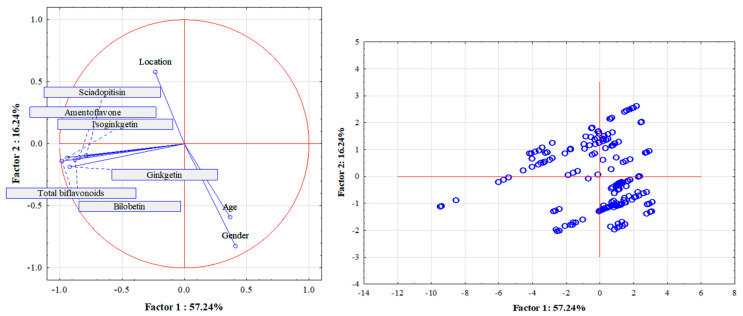
PCA (**left**) biplot and (**right**) score plot of the selected variables and experimental results.

**Figure 5:**
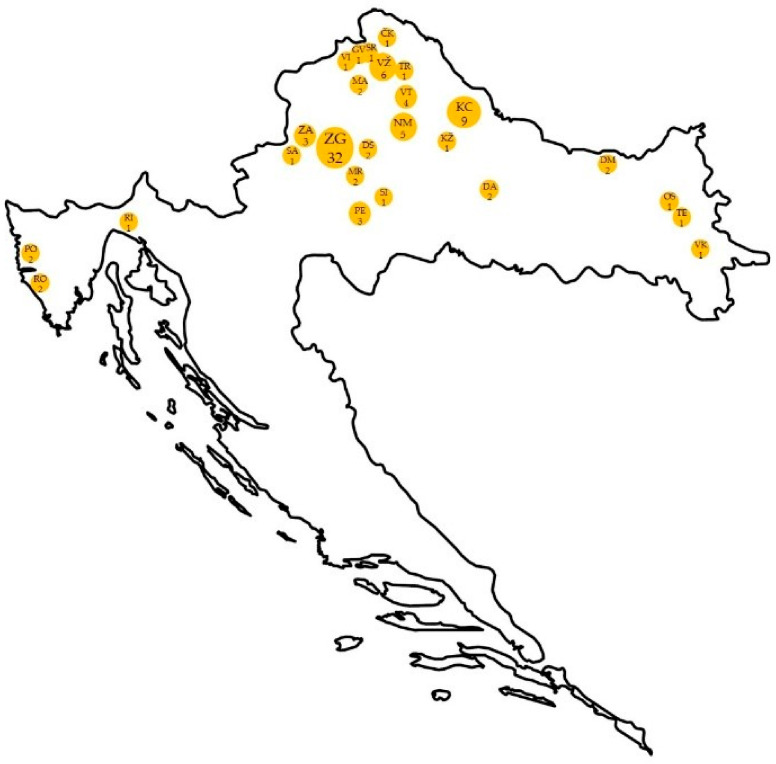
Locations of sampled trees and the number of trees sampled at each location.

To statistically evaluate the factors that may influence biflavonoid content in our samples, we performed principal component analysis and modeled the relationships between location, tree age, gender, and the biflavonoid profile. Figure 3 presents the Spearman correlation matrix summarizing the relationships among location, age, gender, individual biflavonoids (amentoflavone, bilobetin, ginkgetin, isoginkgetin and sciadopitysin), and total biflavonoids. Overall, the matrix reveals clear patterns of association, with strong positive correlations among most biflavonoids and comparatively weak or negative correlations with location, age and/or gender. The strongest relationships are observed among the biflavonoids themselves. Total biflavonoids show very high positive correlations with ginkgetin (r = 0.928), isoginkgetin (r = 0.930), bilobetin (r = 0.911), sciadopitysin (r = 0.888), and amentoflavone (r = 0.792). This trend is expected because all individual biflavonoids contribute to the overall total biflavonoid content. Strong intercorrelations are evident between bilobetin and isoginkgetin (r = 0.942), ginkgetin and sciadopitysin (r = 0.853), and amentoflavone with both ginkgetin (r = 0.715) and isoginkgetin (r = 0.759). In contrast, location, age and/or gender show weaker and often negative associations with biflavonoids measures what is also evident from above Figure 4 and Figure 5. Age is moderately negatively correlated with most biflavonoids, particularly ginkgetin (r = −0.275), isoginkgetin (r = −0.289), and total biflavonoids (r = −0.328), suggesting a general decline in biflavonoids expression with increasing age category. Gender also shows weak negative correlations with individual biflavonoids and total biflavonoids (r = −0.113), while location exhibits minimal correlations overall, indicating limited spatial influence on the measured biflavonoids.

PCA was employed to reduce dimensionality and visualize the underlying structure of the 90-specimen dataset (Figure 4). The first two principal components (PCs) accounted for 73.48% of the total variance (Table 2). Factor 1 explains 57.24% of the total variance, while Factor 2 accounts for 14.24%, together capturing over 71% of the variability in the dataset. Most biflavonoids variables load strongly on Factor 1, indicating that this axis represents a general biflavonoids gradient. Total biflavonoids, bilobetin, ginkgetin, isoginkgetin, and amentoflavone cluster closely and point in a similar direction, confirming their strong positive interrelationships observed in the correlation analysis. Sciadopitysin and location load more strongly on Factor 2, suggesting that this component reflects variation associated with spatial or contextual influences rather than overall biflavonoids intensity. Age and gender vectors point in a different direction from the main biflavonoids cluster, indicating weaker and partially opposing relationships with biflavonoids traits. The proximity and angles between vectors highlight positive associations among biflavonoids indicators and weaker, more independent contributions of demographic variables. Overall, the biplot confirms a coherent behavioral structure dominated by Factor 1, with secondary variation linked to location-related effects. The eigenvalue distribution shown in Table 2 further supports this interpretation. While Factors 1 and 2 exceed or approach the commonly used Kaiser criterion (eigenvalue > 1), subsequent components contribute progressively smaller proportions of variance. Factor 3 explains 11.97% of the variance, increasing the cumulative explained variance to 85.45%, whereas Factors 4 to 8 each account for less than 7.1% individually. The sharp decline in eigenvalues after the second component indicates diminishing explanatory power, reinforcing the dominance of the first two factors in structuring the dataset.

Furthermore, the score plot of the experimental results (Figure 4 left) shows that the samples are mainly dispersed along Factor 1, indicating that this component represents the dominant source of variation among observations, most likely related to overall behavioral intensity. A noticeable clustering of samples according to total biflavonoids concentration can be observed, with many points grouped near the centre of the plot, suggesting a high degree of similarity among experimental results. However, several samples are spread toward both positive and negative extremes of Factor 1, reflecting heterogeneity in behavioral responses. Factor 2 contributes additional separation, allowing partial discrimination of samples that overlap along Factor 1. The absence of clearly isolated clusters indicates gradual transitions rather than distinct groups. Overall, the score plot supports the presence of continuous variation in the experimental data, consistent with the correlated behavioral patterns identified in the PCA loading and correlation analyses.

Table 3 summarizes the parameters and performance statistics of the piecewise linear regression (PLR) models developed to evaluate individual and total biflavonoid contents as a function of location (*X*_1_), age (*X*_2_), and gender (*X*_3_). For each output variable, the table reports the model equations defined over two response ranges, together with the coefficient of determination (*R*^2^), adjusted *R*^2^ (*R*^2^_adj_), and root mean square error (*RMSE*), providing a comprehensive evaluation of explanatory power.

Overall, the PLR models exhibit good to strong evaluation performance, with R^2^ values ranging from 0.7468 to 0.8304, indicating that approximately 75–83% of the variability in individual and total biflavonoid concentrations can be explained by the selected predictors. The close agreement between *R*^2^ and *R*^2^_adj_ across all models suggests that the inclusion of location, age, and gender is justified and does not lead to overfitting, reinforcing the robustness of the model structure.

For amentoflavone (*Y*_1_), the model achieves an *R*^2^ of 0.8067, indicating a strong fit. In the lower concentration range (*Y*_1_ ≤ 55.5762), age shows a positive contribution, while location and gender exhibit negative effects. In contrast, at higher concentrations (*Y*_1_ > 55.5762), all predictors display larger coefficients, with location and age exerting strong positive influences and gender having a pronounced negative effect. This shift in coefficients between regimes suggests non-linear behavior in amentoflavone accumulation, potentially reflecting changes in biosynthetic regulation across concentration levels. The *RMSE* value of 15.29 indicates relatively low error compared with the observed concentration range. Bilobetin (*Y*_2_) is described with an *R*^2^ of 0.7588 and an *R*^2^_adj_ of 0.7561, demonstrating satisfactory explanatory capacity. In the lower response range, age negatively influences bilobetin levels, while location and gender contribute positively. In the higher range (*Y*_2_ > 406.6047), the magnitude of all coefficients increases substantially, particularly for age and gender, indicating stronger demographic effects at elevated concentrations. The higher *RMSE* (63.25) reflects the broader variability of bilobetin concentrations relative to amentoflavone. The ginkgetin (*Y*_3_) and isoginkgetin (*Y*_4_) models display comparable performance, with *R*^2^ values of 0.7468 and 0.7732, respectively. For both compounds, age consistently shows a negative association, especially pronounced in the higher concentration regimes, while location tends to have a positive influence. Gender effects vary between the two concentration ranges, suggesting complex interactions between demographic factors and compound-specific biosynthesis. The *RMSE* values for ginkgetin (154.78) and isoginkgetin (160.97) are higher than those for the less abundant compounds, consistent with their larger absolute concentration ranges and increased dispersion. Sciadopitysin (*Y*_5_) exhibits the strongest model performance, with an *R*^2^ of 0.8304 and *R*^2^_adj_ of 0.8285, indicating excellent evaluation capability. In the lower concentration range, all predictors contribute positively, highlighting a cumulative effect of location, age, and gender. However, at higher concentrations, age and gender switch to negative coefficients, while location remains positively associated. This pattern suggests that environmental or spatial factors become increasingly dominant in determining sciadopitysin levels at elevated concentrations. The *RMSE* of 229.20 reflects the high variability inherent to this compound.

Finally, the model for total biflavonoids (*Y*_6_) integrates the combined effects observed for individual compounds and yields an *R*^2^ of 0.7636. Location and gender show strong positive effects, particularly in the higher concentration regime, whereas age consistently exerts a negative influence, indicating a potential decline in total biflavonoid accumulation with increasing age. The *RMSE* of 107.97 demonstrates acceptable evaluation accuracy for an aggregate response variable. Overall, the obtained results demonstrate that the PLR approach effectively captures both linear and regime-dependent relationships between biflavonoid concentrations and demographic predictors. The consistent explanatory strength, low discrepancy between *R*^2^ and *R*^2^_adj_, and reasonable *RMSE* values confirm the suitability of the models for describing individual and total biflavonoids while highlighting meaningful variations in predictor influence across concentration ranges.

## 3. Discussion

In our study, data related with the presence of biflavonoid in ginkgo leaves is in accordance with literature data. Similarly, Lei et al. [27], Beck and Stengel [11], and Wang et al. [28] reported the presence of bilobetin, ginkgetin, isoginkgetin, and sciadopitysin in ginkgo leaves, while amentoflavone was not detected. In most of our samples, as well as in the average values, a clear trend in biflavonoid concentrations was observed: amentoflavone < bilobetin < ginkgetin < isoginkgetin < sciadopitysin, which is consistent with literature data [14,28]. Many studies note challenges in separating the stereoisomers ginkgetin and isoginkgetin, which are sometimes reported as a single compound [11,16]. In our analysis, these two compounds were well separated, and on average we detected slightly higher levels of isoginkgetin than ginkgetin. Our results indicate that sciadopitysin was present at the highest average concentration, approximately 1.5 mg/g dw, consistent with previous studies reporting sciadopitysin as the predominant biflavonoid in ginkgo leaves [14,15,29,30,31]. Some authors have reported isoginkgetin as the predominant biflavonoid in ginkgo leaves [27,32]. Interestingly, in our previous study, we reported sciadopitysin as the predominant biflavonoid in ginkgo leaf blades, whereas in petioles we detected higher levels of isoginkgetin and bilobetin than sciadopitysin [30].

This similarity in the observed pattern is also supported by principal component analysis, which revealed a coherent overall structure in the data, with secondary variation associated with location-related effects. Since most of the trees from which we collected samples grow in similar climatic regions and at low altitudes, variations in biflavonoid levels are likely influenced by local microenvironmental conditions, such as soil properties, sunlight exposure, and moisture availability. This location effect is expected, as the presence of specific specialized metabolites, such as biflavonoids in ginkgo, is genetically determined, while their concentrations may be influenced by environmental factors [18]. Kaur et al. [16] analyzed ginkgo samples collected across a range of altitudes (600–1710 m) and found that flavonoid and biflavonoid content did not follow a consistent trend, likely due to microlocation factors, similar to those influencing our samples.

PLR models generally indicate a potential decline in total biflavonoid accumulation with increasing age, although this trend is not consistent across individual biflavonoids. Specifically, age has a strong positive effect on amentoflavone, a negative effect on bilobetin, and a consistently negative association with ginkgetin and isoginkgetin. For sciadopitysin, age contributes positively at lower concentrations, but at higher concentrations, the effect becomes negative. Our results are in accordance with literature data where is established that in ginkgo younger leaves accumulate higher levels of monomeric flavonoids; therefore, for pharmaceutical purposes, young ginkgo leaves are typically used [21]. Pandey et al. [15] compared biflavonoid content in leaves of trees aged 175, 84, and 8 years and reported higher sciadopitysin levels in the oldest tree. However, their study was based on a very small sample size (one tree per age), whereas our study includes 90 trees, and differences in their results may also be influenced by additional environmental factors since our data also shows that at higher sciadopitysin level also other microlocation factors may influence their level.

According to the PLR analysis, gender effects also vary depending on the analysed compounds and concentration range, suggesting complex interactions between demographic factors and compound-specific biosynthesis. At lower concentrations, the tree’s biflavonoid levels are primarily limited by external factors (soil, light, location). The model shows smaller coefficients here because the plant has the capacity to produce more if the environment allows. Once the concentration passes the breakpoint (e.g., 1531 µg/g for sciadopitysin), the plant reaches metabolic limit. At this stage, internal biological constraints, specifically age, become the dominant factor. This explains why the PLR coefficients for age (β) jump from a small negative number to a very large negative number after the breakpoint. This is in accordance with literature data on flavonoid content in ginkgo leaves in relation to tree gender which are inconsistent. Pandey et al. [15] reported higher levels of sciadopitysin in male leaves than in female leaves; however, their study was based on only one tree of each sex, and the observed differences may therefore have been influenced by microlocation factors. For example, Koczka et al. [33] determined total polyphenol and flavonoid content in leaves collected from male and female ginkgo trees and reported higher levels in leaves from male trees. Similarly, Liao et al. [34] analyzed flavonoid content and the expression of genes related to flavonoid biosynthesis in 50 male and 50 female ginkgo samples. Their results showed that all flavonoid biosynthesis genes, from flavanone 3′-hydroxylase (F3′H) to anthocyanidin reductase (ANR) and leucoanthocyanidin reductase (LAR), exhibited higher expression levels, to varying degrees, in female saplings than in male saplings. In contrast, Gao et al. [35] analyzed flavonoids in male and female ginkgo seedlings and found higher concentrations in female seedlings. Overall, these results highlight the complexity of factors influencing flavonoid biosynthesis, particularly biflavonoids, in ginkgo leaves. Both intrinsic factors, such as age and gender, and extrinsic factors, such as location and microenvironmental conditions, interact in a compound-specific manner, indicating that biflavonoid accumulation is shaped by a dynamic interplay of genetic, developmental, and environmental influences.

## 4. Materials and Methods

### 4.1. Chemicals, Reagents, and Instruments

HPLC-grade biflavonoid standards (amentoflavone, ginkgetin, isoginkgetin, bilobetin, and sciadopitysin) were obtained from PhytoLab (Vestenbergsgreuth, Germany). Mobile phases were prepared using acetonitrile, LC gradient grade (LiChrosolv^®^, Merck, Darmstadt, Germany), formic acid (98–100%, Sigma-Aldrich, Darmstadt, Germany), and ultrapure water (ELGA LabWater, Wycombe, UK). Plant material was ground and homogenized using a knife mill (POWTEQ HM100, Beijing, China). Powdered samples were accurately weighed using an analytical balance (Adam Equipment, Maidstone, UK). Extractions were performed sequentially using a vortex mixer (V-1 Plus, Biosan, Riga, Latvia), an ultrasonic bath (DU-100, Argo Lab, Carpi, Italy), and a mechanical rotator (Bio RS-24, Biosan, Riga, Latvia). Supernatants were separated by centrifugation using a centrifuge (LMC-4200R, Biosan, Riga, Latvia). Biflavonoid identification and quantification was performed using an Agilent 1260 Infinity II HPLC system equipped with a diode array detector (DAD) (Agilent, Santa Clara, CA, USA).

### 4.2. Plant Material

Ginkgo (*Ginkgo biloba* L.) leaves were collected between 20 and 30 of October 2024. According to our previous study [13] minimal changes in biflavonoid content occur in autumn leaves in that period. From each tree, 30 leaves were harvested at a height of approximately 2.0–2.5 m above ground level. The collected leaves were air-dried at room temperature. Air-drying of ginkgo leaves yields biflavonoid contents comparable to those obtained by freeze-drying [36]. A voucher specimen was deposited at the University North herbarium, including detailed information on the collection location and specimen number.

In total, ginkgo leaf samples were obtained from 90 individual trees across the Republic of Croatia. All ginkgo trees were located in landscaped areas, including city parks, parks surrounding religious sites, private gardens, or along urban streets, and were all cultivated for their ornamental value. The geographic distribution of the sampled trees is shown in Figure 5. Most of the locations, except for two in Istria and one in Rijeka, are in the continental part of Croatia, which has a temperate continental climate, and the samples were collected at low altitudes, up to a maximum of 250 m above sea level.

**Figure 6:**
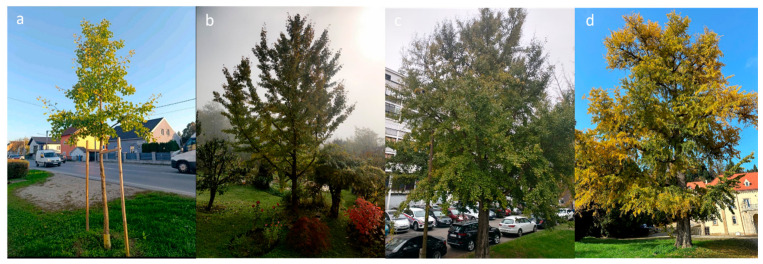
Examples of ginkgo trees in different age categories: younger than 10 years (**a**), 10–30 years (**b**), 30–100 years (**c**), and older than 100 years (**d**).

Trees are classified by age into four categories, younger than 10 years, from 10 to 30 years, from 30 to 100 years and older than 100 years. The examples of tree from each age categories are shown at Figure 6.

Gender of the trees was determined by the presence or absence of female reproductive structures. Since ginkgo trees typically reach sexual maturity at around 20–30 years of age, only trees older than 30 years were included in the analysis of sex-related effects.

### 4.3. Extraction

Air-dried plant material was processed into a fine, homogeneous powder (particals size < 300 µm) using a knife mill. For extract preparation, 30 mg of the powder was accurately weighed in triplicate, and 1 mL of 70% ethanol was added to each sample, followed by brief vortexing to homogenize the mixture. Extraction was initially performed by sonication in an ultrasonic bath (40 kHz) for 10 min, after which the samples were rotated for 45 min on a mechanical rotator to ensure uniform extraction. The suspensions were then centrifuged at 3800 rpm for 5 min, and the resulting clear supernatants were filtered through 13 mm, 0.4 µm hydrophobic PTFE syringe filters prior to HPLC analysis.

### 4.4. Biflavonoid Profiling

For the identification and quantification of biflavonoids, we used methods previously published by Medvedec et al. [37]. Data acquisition and processing were performed using Agilent OpenLAB CDS software (version 2.6). Biflavonoids were identified by comparing sample spectra with commercially available reference standards, and chromatograms were recorded at 330 nm. The concentrations of five biflavonoids in the samples were quantified using standard calibration curves and expressed as µg/g dry weight (dw). Total biflavonoid content was calculated as the sum of all quantified biflavonoids.

### 4.5. Statistical Analysis and Mathematical Modeling

#### 4.5.1. Basic Statistical Analysis and Correlation Matrix

Descriptive statistics, including means, standard deviations, and distribution normality tests, were calculated using the Statistica 14.0 software package (Tibco Software Inc., Santa Clara, CA, USA). The correlations between the experimental data were also analysed using the Spearman correlation matrix in Statistica 14.0. Before conducting the correlation analysis, all non-numerical variables (location, age group, and gender) were transformed into numerical codes to ensure compatibility with statistical procedures. Categorical data cannot be directly processed in correlation matrices, so each category was assigned a unique numerical value using standard coding procedures. For example, gender categories (no defined, male, female) were represented by numbers 0–2, while different locations were encoded as separate numerical identifiers (1–27). Age, originally recorded in non-numerical form (<10 years was coded with 1; 10–30 years with 2, 30–100 years with 3, and >100 years with 4), was converted into ordered numerical categories to preserve its ordinal nature. This coding process enabled the inclusion of these variables in multivariate analyses and ensured that the correlations could be accurately computed and interpreted.

#### 4.5.2. Principal Component Analysis

To elucidate the internal structure of the dataset and reduce dimensionality, Principal Component Analysis (PCA) was performed on the standardized autoscaled data. PCA transformed the correlated biflavonoid concentrations into a set of linearly independent principal components (PCs). The primary sources of variance were identified using the Kaiser criterion (eigenvalues > 1) and visualized via biplots to determine the influence of demographic factors on the biflavonoid gradient. All PCA analyses were conducted in Statistica 14.0 (Tibco Software Inc., Santa Clara, USA) using the standard multivariate analysis parameters.

#### 4.5.3. Modeling the Correlation Between Location, Age and Gender of the Tree and Biflavonoids Profile

The influence of location (*X*_1_), age (*X*_2_), and gender (*X*_3_) on individual and total biflavonoid concentrations (*Y*) was quantified using Piecewise Linear Regression (PLR). This approach accounts for non-linearities in metabolite accumulation by establishing distinct regimes around an optimized breakpoint (*b_n_*). This relationship is described by equations:

$$concentration\;of\;individual\;and\;total\;biflavonoids=f\left(location,\;age,\;gender\right)$$

$$Y=\left(b_{01}+b_{11}\cdot X_{1}+b_{21}\cdot X_{2}+b_{31}\cdot X_{3}\right)\;\left(for\;Y\leq b_{n}\right)\;+\left(b_{02}+b_{12}\cdot X_{1}+b_{22}\cdot X_{2}+b_{32}\cdot X_{3}\right)\;\left(for\;Y>b_{n}\right)$$

Model parameters were estimated using the Levenberg–Marquardt algorithm, employing a quasi-Newton optimization procedure to minimize the global sum of squared residuals. The iterative process was conducted with a convergence criterion of 10^−6^ over 50 iterations. Model robustness and evaluation accuracy were evaluated using the coefficient of determination (*R*^2^), adjusted *R*^2^, and the Root Mean Square Error (RMSE) at a 95% confidence interval.

## 5. Conclusions

This study provides the most extensive spatiotemporal characterization of the ginkgo biflavonoid profile to date, utilizing a population-scale approach across 90 individual trees. Our results confirm a highly conserved biosynthetic hierarchy (sciadopitysin > isoginkgetin > ginkgetin > bilobetin > amentoflavone), yet reveal significant heterogeneity driven by the interplay of ontogeny and microenvironment Principal component analysis revealed a coherent overall structure in the biflavonoid profile, while secondary variation was associated with location and microenvironmental conditions. Partial least squares regression models indicated that age and gender affect biflavonoid accumulation in a compound-specific manner: for example, age positively influences amentoflavone at lower concentrations but negatively affects ginkgetin, isoginkgetin, and bilobetin, whereas gender effects vary depending on the compound and concentration range. Overall, these results demonstrate the complex interplay of intrinsic (genetic, age, and gender) and extrinsic (location, microenvironment) factors in determining biflavonoid content, emphasizing that both developmental stage and environmental context must be considered when evaluating ginkgo leaf chemistry, particularly for pharmaceutical applications. In the future, larger studies should be conducted that integrate both metabolomic and transcriptomic analyses, combined with modern data processing approaches, to more comprehensively understand the regulation and biosynthesis of biflavonoids in ginkgo leaves.

However, it should be noted that this study is based on a defined number of sampling sites and individual trees within a specific geographic region; therefore, the conclusions regarding environmental and location effects should be interpreted within this context. While the present results are directly supported by our dataset, broader generalizations remain hypotheses that require further validation. Future studies should therefore include a larger number of populations, additional plant species, and ideally a global-scale sampling design encompassing trees from different climatic regions to confirm the observed patterns and improve their general applicability.

## Figures and Tables

**Table 1:** Maximum, minimum, mean values, standard deviations, and coefficient of variation of the individual biflavonoids and total biflavonoid content.

	Detected	Maximum µg/g dw	Minimum µg/g dw	Mean µg/g dw	Standard Deviation µg/g dw	Coefficient of Variation (%)
amentoflavone	61/90	278.62	0	55.13	52.31	94.88
bilobetin	90/90	1925.41	96.41	406.48	332.96	81.91
ginkgetin	90/90	2618.33	83.35	844.49	389.85	46.16
isoginkgetin	90/90	3470.76	267.57	993.21	586.65	59.07
sciadopitysin	90/90	3325.53	572.42	1532.89	544.13	35.49
total biflavonoids		11,517.60	1314.74	3824.57	1708.15	44.66

**Table 2:** PCA eigenvalues.

Value Number	Eigenvalue	Total Variance (%)	Cumulative Eigenvalue	Cumulative (%)
1	5.1512	57.236	5.1512	57.24
2	1.4619	16.243	6.6131	73.48
3	1.0777	11.974	7.6908	85.45
4	0.6364	7.071	8.3271	92.52
5	0.3046	3.385	8.6318	95.91
6	0.1842	2.047	8.8160	97.96
7	0.1552	1.724	8.9711	99.68
8	0.0289	0.321	9.0000	100.00

**Table 3:** Parameters of the PLR model used for evaluation of individual and total biflavonoids based on the location (*X*_1_), age (*X*_2_) and gender (*X*_3_).

Output Variable	Model Equation	*R* ^2^	*R* ^2^_adj_	*RMSE*
Amentoflavone	*Y*_1_ = (40.7072 − 0.5472 · *X*_1_ + 0.7135 · *X*_2_ − 2.1724 · *X*_3_) (for *Y*_1_ ≤ 55.5762) + (4.1118 + 4.2333 · *X*_1_ + 7.7811 · *X*_2_ − 24.3180 · *X*_3_) (for *Y*_1_ > 55.5762)	0.8067	8.045	15.2906
Bilobetin	*Y*_2_ = (248.1895 + 0.8379 · *X*_1_ − 5.8482 · *X*_2_ + 10.823 · *X*_3_) (for *Y*_2_ ≤ 406.6047) + (376.2666 + 30.7322 · *X*_1_ − 146.469 · *X*_2_ + 161.7217 · *X*_3_) (for *Y*_2_ > 406.6047)	0.7588	0.7561	63.2546
Ginkgetin	*Y*_3_ = (543.5706 + 3.3295 · *X*_1_ − 3.4294 · *X*_2_ + 33.8764 · *X*_3_) (for *Y*_3_ ≤ 854.2631) + (1302.7770 + 8.3896 · *X*_1_ − 112.7690 · *X*_2_ − 31.3087 · *X*_3_) (for *Y*_3_ > 854.2631)	0.7468	0.7439	154.7778
Isoginkgetin	*Y*_4_ = (641.2271 + 2.1241 · *X*_1_ − 5.4511 · *X*_2_ + 7.3959 · *X*_3_) (for *Y*_4_ ≤ 993.1492) + (1587.680 + 19.1149 · *X*_1_ − 178.750 · *X*_2_ − 41.4694 · *X*_3_) (for *Y*_4_ > 993.1492)	0.7732	0.7706	160.9703
Sciadopitysin	*Y*_5_ = (1021.4340 + 4.0841 · *X*_1_ + 11.2364 · *X*_2_ + 25.9846 · *X*_3_) (for *Y*_5_ ≤ 1531.1590) + (2027.8210 + 17.3692 · *X*_1_ − 143.527 · *X*_2_ − 15.7391 · *X*_3_) (for *Y*_5_ > 1531.1590)	0.8304	0.8285	229.1996
Total	*Y*_6_ = (1021.4340 + 8.1382 · *X*_1_ − 10.3167 · *X*_2_ + 112.0245 · *X*_3_) (for *Y*_6_ ≤ 3821.9510) + (5255.0910 + 67.4664 · *X*_1_ − 566.718 · *X*_2_ + 101.3535 · *X*_3_) (for *Y*_6_ > 3821.9510)	0.7636	0.7609	107.9707

## Data Availability

The data presented in this study are available on request from the corresponding author.
